# Cell autonomous Vegf-C/Vegfr3 signaling in adult neural stem cells

**DOI:** 10.18632/oncotarget.6325

**Published:** 2015-11-13

**Authors:** Tae Hyuk Kang, Jinah Han, Jean-Leon Thomas

**Affiliations:** Universite' Pierre and Marie Curie- Paris 6, INSERM/CNRS U-1127/UMR-7225, 4APHP, Groupe Hospitalier Pitié-Salpètrière, Paris, France

**Keywords:** neural stem cell activation, vascular endothelial growth factor signaling, Vegf-C, Vegfr3

In the adult mammalian brain, the potential to generate new neurons is restricted to a limited number of sites, called neurogenic niches, which are mainly the subventricular zone lining the cerebral ventricles and the dentate gyrus of the hippocampus. Neurogenic niches harbor a unique population of neural stem cells (NSCs), self-renewing cells with hallmarks of astrocytes that generate neurons and glia. Brain injury in humans as a result of trauma, hypoxia or neuron-glial pathologies activates neurogenesis in these niches, attesting to an endogenous repair potential that is, however, generally not sufficient to allow complete rescue of lost tissues. To safely enhance this endogenous neurogenic response, it is crucial to characterize mechanisms controling NSCs in their niche.

NSCs are predominantly found in a quiescent state but can be activated to either self-renew or generate neural progenitors cells (NPCs). Understanding NSC activation mechanisms is a major goal in NSC biology and a variety of synergetic and antagonistic factors have been shown to dynamically regulate NSCs in each neurogenic niche [1]. The majority of signals originate from NSC-neighboring cells such as ependymal, endothelial, microglial or neuronal cells [2]. However, adult NSCs themselves also contribute to NSC activation and niche homeostasis through autocrine signaling [1]. We and others have recently underscored a specific role for Vascular endothelial growth factors (Vegfs) in these processes [3-6].

Our studies have focused on the expression and role of Vegf-C, the main lymphangiogenic factor, and Vegfr3, the high affinity receptor of Vegf-C, in adult neurogenic niches [4, 5]. First, we found that Vegf-C displays an enriched expression in NSCs compared to neighboring cells of adult niches. Similarly, Vegfr3 is expressed by NSCs while its expression declines in NPC progeny. Second, conditional *Vegfr3* deletion in NSC, using the *Glast^CreERT2^; Vegfr3^lox^* mouse model, decreases the number of dividing NSCs/NPCs and newborn neurons, without affecting the pool of NSCs. Vegfr3 activity is therefore not necessary for NSC maintenance, but is required for the generation of progenitors and neuroblasts. Third, *in vitro* and *in vivo* approaches revealed that Vegf-C treatment increased the number of dividing cells in cultured NSCs and neurogenic niches, respectively. In contrast to Vegf-A [7], another closely related vascular growth factor expressed in neurogenic niches, Vegf-C activates NSCs without inducing vascular proliferation. Thus, Vegf-C may function more specifically as an activator of NSCs in neurogenic niches. Using transgenic *Vegfr3^YFP^* reporter mice that allow isolation of Vegfr3-expressing NSCs by FACS from neurogenic niches, we have characterized the transcriptomic profile of NSCs, as well as their reponse to Vegf-C. NSCs treated with Vegf-C lose expression of NSC markers while increase expression of progenitor cell markers and cell cycle activation genes. Therefore, Vegf-C/Vegfr3 signaling promotes both division and differentiation of NSCs in a cell-autonomous manner.

In endothelial cells, Vegf-C/Vegfr3 signaling is known to control proliferation and survival through activation of Akt and Erk intracellular cascades. To address whether these molecular mechanisms were conserved in NSCs, we performed gene set enrichment tests on Vegfr3-expressing NSCs and selected 1750 genes with significant expression change following Vegf-C stimulation (unpublished data). Signaling network analysis using Reactome FIViz showed that the initial signaling cluster induced by Vegf-C stimulation included focal adhesion, PI3K-Akt, Rap1, Ras, Vegfr3 and β1 integrin signaling pathways. Pathway interaction analysis revealed that Akt1, 2 and 3 are the common effectors of these signaling pathways and suggested that the PI3K-Akt signaling may be the convergent down-stream target of Vegf-C/Vegfr3 signaling in NSCs. Further biochemical analysis in human embryonic stem cell-derived NSCs revealed that expression of Vegfr3 in NSCs was conserved between mouse and human. In addition, Vegf-C treatment rapidly induced Vegfr3 phosphorylation as well as phosphorylation of Akt (T308 and S473) and Erk, similarly to endothelial cells. Taken together, these data suggest that Vegf-C/Vegfr3 intracellular signaling pathways are conserved between NSCs and endothelial cells.

In conclusion, the discovery of Vegf-C/Vegfr3 as a ligand-receptor signaling system specific for NSC activation in adult neurogenic niches paves the way for the establishement of Vegf-C-based therapies to enhance neurogenesis during aging and to improve the efficacy of NSC-based repair therapies in patients with neurodegenerative diseases.
